# Protective role of the half-center oscillator connectivity against external perturbations

**DOI:** 10.1186/1471-2202-14-S1-P77

**Published:** 2013-07-08

**Authors:** William Barnett, Aaron Gomez-Lugo, Gennady Cymbalyuk

**Affiliations:** 1 Neuroscience Institute, Georgia State University, Atlanta, Georgia 30303, USA

## 

Central pattern generators (CPGs) are neuronal circuits that control rhythmic movements in animals. The heartbeat of the medicinal leech is controlled by a CPG that is distributed over several ganglia. In ganglia 3 and 4, there are half-center oscillators (HCOs): pairs of reciprocally inhibitory heart interneurons (HNs). These HCOs form the kernel of the heartbeat CPG, and each HCO is composed of a pair of endogenously bursting heart interneurons [1]. A canonical model of the HN accurately reproduces experimental results [1,2]. It also exhibits bistability of bursting and silence in single HNs and the HCO [1,3]. The single HN and HCO model have been expanded into a family of models that satisfy biological constraints [2]. Multistability is highly prevalent in this database [4]. The coexistence of bursting and silent regimes could present a life threatening condition for the animal. If a perturbation were able to trigger a switch in the activity of the CPG to a silent state, circulation of blood in the leech would be crippled. Our hypothesis is that the HCO connectivity protects functional pattern of activity against an external perturbation.

**Figure 1:**
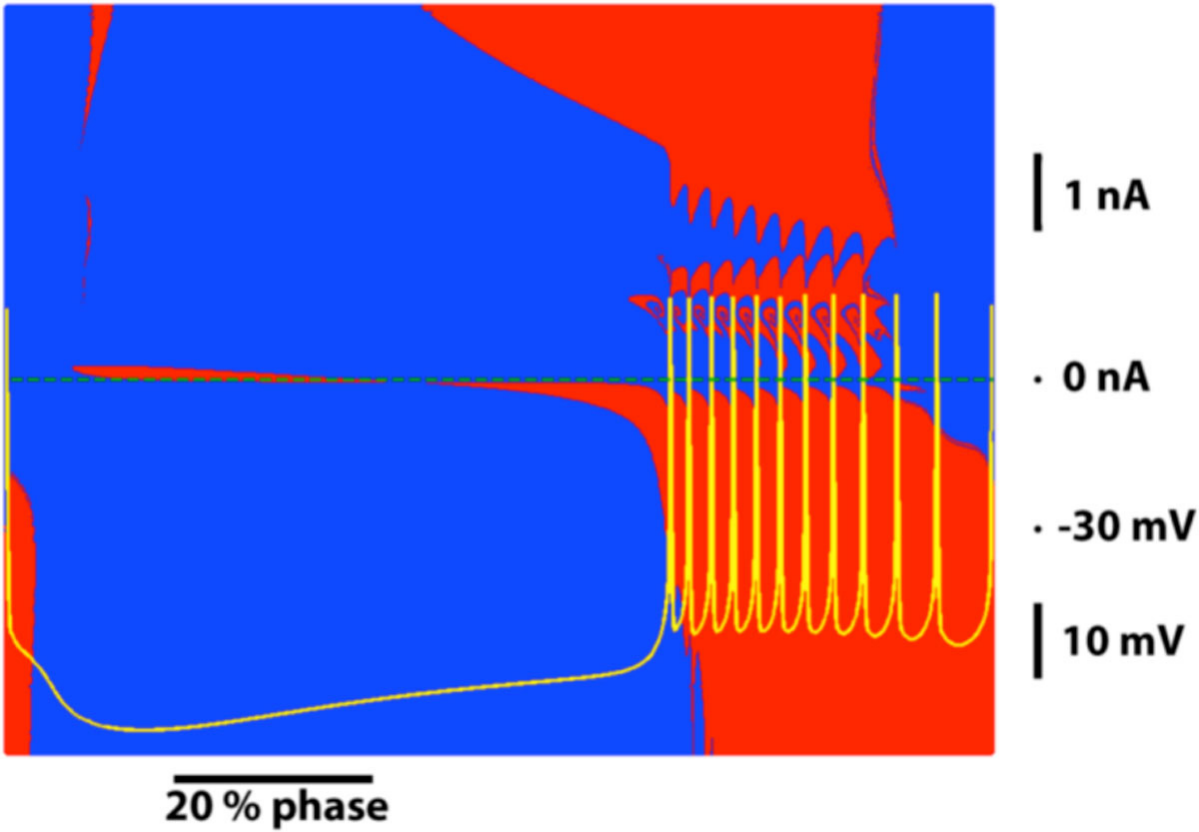
**Properties of pulses triggering a switch from bursting to silence**. Red or blue points indicate a switch or failure to switch. The yellow trajectory is the bursting waveform that defines the phase. The green dashed line indicates 0 nA.

Here, we investigated the perturbations that switch the activity of the HN and the HCO from bursting to silence. Over a series of simulations, we applied a 30 ms pulse of current with various amplitudes and phases of application in the period of bursting activity. This protocol provided a set of pulse parameters in the (phase, amplitude) parameter space for which a switch was triggered. In the canonical model of a single HN for which the leak conductance was 9.59 nS, both depolarizing and hyperpolarizing pulses could trigger switches (Figure 1). We observed contiguous sets of pulse parameters where triggered switches occurred, but there were also fine structures in the parameter space (Figure 1). We estimated the susceptibility of bursting activity in the HN to perturbations by the proportion of pulse parameter sets that would trigger a switch to the total number of trials. We found that 20.84% of pulses triggered a switch (Figure 1). We repeated this pulse protocol in the HCO for the same parameter values of the ionic conductances, and found that no pulses elicited a switch from bursting to silence. Our results suggest that in a network consisting of bistable elements, the half-center oscillator connectivity protects the functional regime against external perturbations.
